# Evidence-based measures to prevent central line-associated bloodstream infections: a systematic review**^1^**

**DOI:** 10.1590/1518-8345.1233.2787

**Published:** 2016-09-01

**Authors:** Daniele Cristina Perin, Alacoque Lorenzini Erdmann, Giovana Dorneles Callegaro Higashi, Grace Teresinha Marcon Dal Sasso

**Affiliations:** 2 MSc. in Nursing Care Management, RN, Hospital Universitário, Universidade Federal de Santa Catarina, Florianópolis, SC, Brazil.; 3 PhD, Full Professor, Departamento de Enfermagem, Universidade Federal de Santa Catarina, Florianópolis, SC, Brazil.; 4 Post-doctoral fellow, Departamento de Enfermagem, Universidade Federal de Santa Catarina, Florianópolis, SC, Brazil.

**Keywords:** Catheter-Related Infections, Central Venous Catheters, Intensive Care Units, Evidence-Based Practice

## Abstract

**Objective:**

to identify evidence-based care to prevent CLABSI among adult patients hospitalized in ICUs.

**Method:**

systematic review conducted in the following databases: PubMed, Scopus, Cinahl, Web of Science, Lilacs, Bdenf and Cochrane Studies addressing care and maintenance of central venous catheters, published from January 2011 to July 2014 were searched. The 34 studies identified were organized in an instrument and assessed by using the classification provided by the Joanna Briggs Institute.

**Results:**

the studies presented care bundles including elements such as hand hygiene and maximal barrier precautions; multidimensional programs and strategies such as impregnated catheters and bandages and the involvement of facilities in and commitment of staff to preventing infections.

**Conclusions:**

care bundles coupled with education and the commitment of both staff and institutions is a strategy that can contribute to decreased rates of central line-associated bloodstream infections among adult patients hospitalized in intensive care units.

## Introduction

Central Venous Catheters (CVC) play an important role in the treatment of hospitalized patients, especially critically ill patients^1^. Intensive Care Units (ICU) employ measures such as diagnostic procedures and invasive devices that may trigger complications such as healthcare-associated infections (HAI)^2^. The challenges imposed to the prevention of nosocomial infections are even greater in an ICU due to the variety of microorganisms, often multiresistant, which require the use of broad-spectrum antibiotics. ICUs are characterized by performing invasive procedures intended for diagnostic purposes or to enable the cure of patients, but which complicate the control of infections^3^. Note that central line-associated bloodstream infection (CLABSI) is the primary complication of central venous catheters^4^.

In the United States, from 250,000 to 500,000 CLABSIs are estimated to occur every year, which result in a rate from 10% to 30% of mortality^5^. A study was conducted in Brazil with 33 patients hospitalized in an adult ICU using a total of 50 CVCs. Of these, 18 were diagnosed with CLABSI. In regard to clinical outcome, 20% of the patients who presented CLABSI died. The incidence of primary bloodstream infection was 1.52/1,000 catheters-day and the CVC utilization rate 0.80^6^. Critical care workers should be aware of CLABSI rates in the ICUs in which they work and devise quality control programs to attain rates not higher than 0.5-1/1,000 CVC/day^7^. 

In this sense, there is a concern over the risk of infections to which patients are exposed, the prevalence of CLABSI, the need to improve care concerning the implantation and maintenance of CVCs, and the adoption of evidence-based measures to ground the care provided by the health staff. Therefore, systematized care defined by evidence-based guidelines confers safety and quality onto the care provided by the intensive care team and can effectively reflect decreased HAI rates.

Seeking to contribute to safer care provided to critically ill patients, this study's aim was to identify evidence-based care to prevent central line-associated bloodstream infection among adult patients hospitalized in intensive care units.

## Method

A systematic review was conducted in accordance with the protocol proposed by the Federal University of São Paulo (UNIFESP), together with Cochrane Brazil, namely: establishing the research question (using the PICO strategy); identifying and selecting studies; critically assessing studies; collecting data; analyzing and presenting data; and interpreting results^8^.

The PICO strategy resulted in the following question: *"What are the CLABSI-related preventive measures implemented among adult patients hospitalized in an ICU?"*

The search was conducted from July 21^st^ to August 10^th^ 2014 in international databases such as Web of Science, Pubmed/Medline, Scopus, Cochrane, Cinahl and Latin American databases, Lilacs/BDENF, through the Coordination of Improvement of Higher Level Personnel (CAPES) platform. The terms used in the search were selected from the MeSH (Medical Subject Headings) as MeSH terms and All Fields, and from DeCS (Health Sciences Descriptors) as descriptors and key words. The Boolean operators AND and OR were used.

The search included studies that answered the research question, were related to the topic, and addressed interventions regarding the care and maintenance of catheters. Inclusion criteria were: original research studies, published from January 2011 to July 2014; written either in Portuguese, English or Spanish; included adult patients; conducted in adult ICUs; included short-term CVCs; and presented abstracts or titles that addressed the subject.

Exclusion criteria were: papers addressing a pediatric or neonatal population; did not originate from research; addressed peripherally inserted central catheter (PICC), hemodialysis, or peripheral and arterial catheters; or did not address preventive measures to prevent central line-associated bloodstream infections.

**Figure 1:**
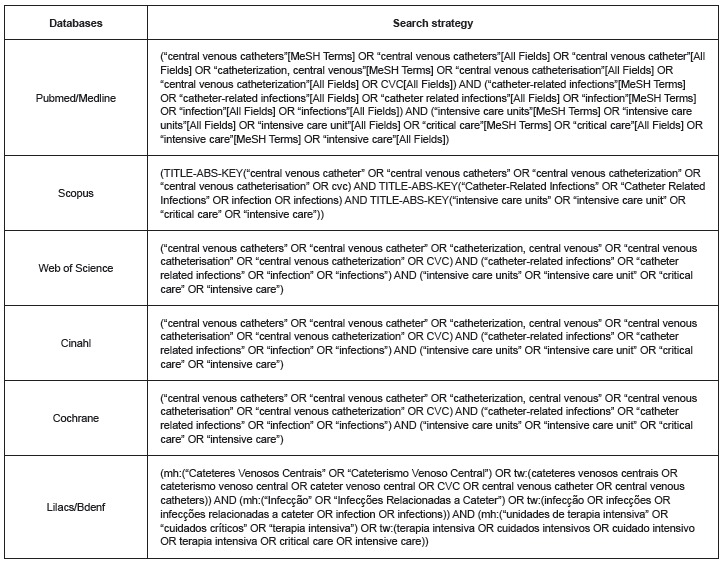
Search strategies used in database search

The search strategy resulted (Figure 1) in 1,611 references, 126 of which were duplicated and were excluded with the help of Mendeley software. A total of 1,485 studies were initially selected, but after reading the titles and abstracts, 1,333 were excluded so that 152 studies remained. Two researchers read the full texts of the 152 studies and any disagreement was discussed until consensus was reached. After this stage, 118 studies were excluded for not meeting the inclusion criteria or because the full text was not available, so that 34 studies were included in this review.

The 34 studies that remained were synthesized and analyzed. They were organized in an analysis tool in Microsoft Word that included Reference, Method, Care, Result and Level of evidence. Data were assessed according to the level of evidence classified by the *Joanna Briggs Institute* ^9^.

## Results

**Figure 2:**
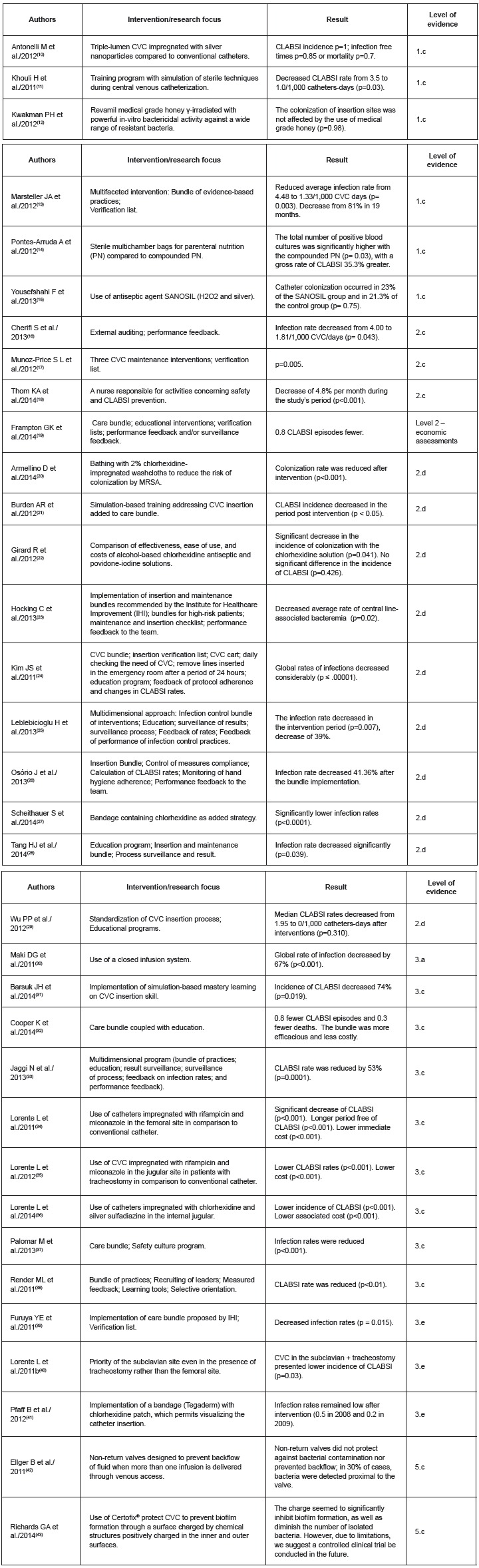
Studies included

This study presents the results with the highest level of evidence concerning measures implemented to prevent CLABSI among adult patients in ICUs. The studies included in the review (Figure 2) tested care bundles, additional interventions beyond established care, and multidimensional interventions addressing both care maintenance and implantation, as well as staff education and institutional interventions.

In regard to the type of study, the following were found: 6 randomized clinical trials (17.6%), 8 cohort studies (23.5%), 10 pre- and posttest studies (29.4%), 3 observational studies (8.8%), 3 quasi-experimental studies (8.8%), 2 systematic reviews (5.8%): one included cohort studies and the other included economic evaluations, and 2 in-vitro tests/bench studies (5.8%).

## Discussion

Nine studies (26.4%) primarily addressed care bundles coupled with education, safety culture or organizational strategies^23^ ^-^ ^24^ ^,^ ^26^ ^,^ ^28^ ^-^ ^29^ ^,^ ^32^ ^,^ ^37^ ^-^ ^39^. One study presented a bundle that contained 5 elements: hand hygiene; maximal sterile barrier precautions during CVC insertion; prepare clean skin with chlorhexidine; avoid the femoral site, if possible; and remove unnecessary catheters. These elements were implemented along with control of measures implementation, collection of information to calculate infection rates, monitoring of adherence to hand hygiene, and feedback of results to the team^26^. Level of evidence: 2.d.

One intervention that focused on quality included two distinct bundles, both contained hand hygiene as the primary element. The first bundle of CVC insertion also contained the following: maximal sterile barrier, prepare skin with chlorhexidine and avoid femoral vein, while the second referred to CVC maintenance: appropriate bandage replacement, aseptic technique to access and change connectors without needles, and daily checking the need of CVC. The intervention was allied with an educational program implemented through conferences and teaching videos, surveillance of process and results^28^. Level of evidence: 2.d.

Following the line of care bundles, one study audited the implementation of a CVC insertion bundle and a maintenance bundle for the Institute for Healthcare Improvement (IHI) associated with checklists and results feedback to the team. The study showed that the medical and nursing approach combined through care bundles reduced the average rate of infection from 6.43 to 1.83^23^. Level of evidence: 2.d.

Another study addressing care bundles presented, in addition to care already mentioned, the use of ultrasound to seek the target vein and confirm intraluminal insertion to reduce complications. The studies achieved marked reduction of global rates of infection with the strategies implemented^24^. Level of evidence: 2.d.

To show the importance of complying with all the elements included in a bundle for it to be effective, one study associated a care bundle proposed by the Institute for Healthcare Improvement (IHI) with a verification list and monitored compliance with the bundle elements. Only 38% reported a high level of compliance. The study emphasizes that only when compliance with a care bundle is high, is it associated with reduced rates of infections^39^. Level of evidence: 3.e.

Three studies (8.8%) presented multidimensional programs that resulted in reduced rates of infections^13^ ^,^ ^25^ ^,^ ^33^. Two studies implemented the multidimensional approach INICC - International Nosocomial Infection Control Consortium, which consists of six simultaneous interventions: bundle of interventions; education; outcome surveillance; process surveillance; feedback on infection rates; and performance feedback on infection control practices ^(^ ^25^ ^,^ ^33^. Level of evidence: 2.c.

A randomized controlled clinical trial tested the multifaceted approach developed by Johns Hopkins Quality and Safety Research Group, which presents evidence-based practices to prevent CLABSI and a program to improve safety, communication, and teamwork. Strategies, such as an intervention team, verification lists, recognition of nurses as potential leaders of interdisciplinary team interventions, data collection to calculate rates, and control of compliance with measures was used. The intervention group achieved a decrease of 81% in non-adjusted CLABSI rates in the 19 months after implementation and the control group achieved a decrease of 69% in 12 months after the intervention. The study emphasized that the role of the nurse as a leader of the multi-professional team was key for the success of interventions ^13^. Level of evidence: 1.c

Four studies (11.7%) addressed educational strategies as the study's main focus, among which two also assessed the cost-effectiveness of this type of intervention^11^ ^,^ ^19^ ^,^ ^21^ ^,^ ^31^. Two studies presented a training program based on the simulation of sterile techniques during CVC insertion and showed that the program decreased infection rates from 3.6 to 1/1,000 catheters-day after the intervention in the first study^11^ (1.c) and the second study reported a decrease of 3.82 to 1.29/1,000 catheters-day^31^. Level of evidence: 3.c.

One study assessed the cost-effectiveness of the strategy previously mentioned, associated with a care bundle, a catheter insertion cart and a verification list as mandatory in the program in which a nurse had the power to interrupt the procedure if the items contained in the list were not complied with. The simulation training was mandatory for all the hospital's physicians and included a pre-course, self-guided reading of papers and instructional books, a 4-hour simulation course supervised by assistant physicians and intensive care workers. The educational strategy resulted in a decrease of 58% in the incidence of CLABSI^21^. Level of evidence: 2.d. One study assessed the efficacy and cost-effectiveness of educational interventions and suggested that a variety of educational approaches could be cost effective and decrease the facility's costs^19^. Level of evidence of economic analysis: 2.

Institutional strategies are considered important when seeking compliance with measures concerning the implantation and maintenance of central catheters. One study focused on external audits to assess compliance with CVC insertion and maintenance practices, presenting monthly feedback to the team. Compliance with care practices increased during the intervention period, showing a significant decrease in the global incidence of infections, though the incidence rate either increased or remained stable after the intervention. The study emphasized the value of auditing- and feedback-based interventions, though reports of lack of leadership and the staff's high turnover represent weaknesses, indicating the need for studies focused on behavioral change strategies^16^. Level of evidence: 2.c.

Extra strategies added to already implemented care concerning the insertion and maintenance of catheters were tested as a means to lower risk of colonization and infection of CVCs^14^ ^,^ ^17^ ^,^ ^27^ ^,^ ^30^ ^,^ ^34^ ^-^ ^36^ ^,^ ^40^ ^-^ ^41^.

Due to the association of CVC with parenteral nutrition (PN), which incurs an increased risk of CLABSI occurring, and seeking to clarify the impact of the infusion system on infection rates, a multi-center study compared sterile multichamber bags for parenteral nutrition (PN). This is considered to be a closed infusion system, with compounded parenteral nutrition (two compounds). The rate of CLABSI was 35.3% greater among patients who received compounded PN in comparison to those who received PN through the closed system^14^. Level of evidence: 1.c.

In regard to bandages impregnated with antiseptic and antibiotics intended to reduce the colonization of bacteria on the catheter insertion site, one study assessed the potential of a bandage containing chlorhexidine to decrease infection. The facility where the study was conducted had already implemented care concerning the insertion and maintenance of catheters, surveillance and education. CLABSI rates were significantly lower among patients using bandages with chlorhexidine, 1.51/1,000 CVS days in comparison to 5.87/1,000 CVC days in patients with conventional bandages^27^. Level of evidence: 2.d.

The influence of different types of catheters on CLABSI prevention and decreasing biofilm formation was addressed in 3 studies^34^ ^-^ ^36^. The use of catheters impregnated with Rifampicin and Miconazole (RM-C) in the femoral site in comparison to standard catheters (SC) showed an incidence significantly lower with the impregnated catheter: 8.61 vs. 0 CLABSI/1,000 catheters-day^34^. Level of evidence: 3.c. Catheters impregnated with Chlorhexidine and Silver sulfadiazine (CHSS) in the internal jugular vein presented lower CLABSI rate than conventional catheters: 0% vs. 2.0%, incidence density of 0 vs. 5.04 CLABSI/1,000 catheters-day^36^. Level of evidence: 3.c.

Three gradual interventions were implemented by a study focusing on the maintenance of catheters in three ICUs: rubbing the insertion site with chlorhexidine swabs for 15s; daily bathing with chlorhexidine-impregnated washcloth; and daily nursing rounds to ensure compliance with the items from a verification list that included infection control measures. The facility where the study was conducted had already been implemented the following list: bandages and catheters impregnated with chlorhexidine or with minocycline/rifampin; skin antisepsis with chlorhexidine; and intravenous connectors without needles. The study reports a progressive decrease in CLABSI rates after the gradual implementation of interventions^17^. Level of evidence: 2.c.

As identified in the bundles presented by the studies, avoiding the femoral site when inserting CVCs is a recommended measure, as well as giving preference to the subclavian vein. One study assessed the use of the subclavian vein in the presence of tracheostomy in comparison to the femoral vein. The "subclavian + tracheostomy" group presented lower incidence of CLABSI when compared to "femoral without tracheostomy", 3.9 vs. 10.0 CLABSI episodes/1,000 catheter days, while there was a tendency for the incidence of CLABSI in the "subclavian + tracheostomy" group to be lower, 3.9 vs. 11.2I CLABSI/1,000 catheters-days^40^. Level of evidence: 3.e.

Studies tested some interventions that did not obtain significant results in reducing infections rates and colonization^10^ ^,^ ^12^ ^,^ ^15^ ^,^ ^18^ ^,^ ^20^ ^,^ ^22^ ^,^ ^42^ ^-^ ^43^. A study investigated whether non-return valves, designed to prevent the backflow of fluids, would be efficacious in reducing infections. The conclusion, however, was that non-return valves do not prevent backflow nor serve as a bacterial filter^42^. Level of evidence: 5.c. One CVC impregnated with silver nanoparticles was assessed, but no significant effect was found and for this reason it cannot be recommended^10^, nor can the use of CVC Certofix^(r)^ Protect (B Braun), which promised to prevent biofilm formation through a charged surface^43^. Level of evidence: 5.c.

In regard to antiseptic solutions to prepare the skin to receive a central venous catheter, a study compared the efficacy, ease of use, and cost of an antiseptic solution with chlorhexidine and a povidone-iodine solution, both alcoholic. The study reports small significant decreases only for the colonization of the catheter and limited ease of use, without significant effects for infection rates or lower cost^22^. Level of evidence: 2.d.

The studies show that actions that include care bundles, the education of workers, the promotion of safety culture, and the implementation of regular assessments controlling compliance with such measures, surveilling infection rates and providing feedback to workers coupled with additional strategies, such as using differentiated catheters and bandages, are important to decrease CLABSI rates among patients hospitalized in adult ICUs.

## Conclusion

This study presents care measures to prevent central line-associated bloodstream infections recently addressed among patients hospitalized in intensive care units. Twenty-six out of the 34 studies analyzed presented significant results concerning decreased central line-associated bloodstream infection rates after the implementation of care. Care measures included with CVC insertion and maintenance to important strategies concerning the staff's education and engagement, safety culture, and surveillance processes.

Nine studies mainly focused on care bundles that included elements such as hand hygiene, cleaning the insertion site with chlorhexidine, avoiding the femoral site, and removing the catheter as soon as it is no longer necessary. Three studies presented multidimensional programs addressing bundles of interventions, education, surveillance, feedback on results, as well as assessment of safety culture, training addressing safety, and partnerships with leaders within the unit.

Three studies addressed educational interventions such as training based on the simulation of sterile techniques. Institutional strategies were also addressed, such as auditing, recruiting of leaders, surveillance, and monthly feedback to the team.

Differentiated care, such as bandages and catheters impregnated with chlorhexidine or antibiotic and closed infusion systems, were also addressed. Eight studies did not present significant results concerning decreased central line-associated bloodstream infection rates like those that tested non-return valves to prevent backflow or catheters using a new antiseptic solution.

This study's limitations include a lack of literature produced in Brazil in the scope of nursing and the fact that the study focuses on only one type of catheter. Studies addressing different types of catheters are important, as are systematic reviews, in order to meet the need of clinical practitioners of implementing evidence-based care.
